# Male C57BL6/N and C57BL6/J Mice Respond Differently to Constant Light and Running-Wheel Access

**DOI:** 10.3389/fnbeh.2019.00268

**Published:** 2019-12-10

**Authors:** Kimberly M. Capri, Marissa J. Maroni, Hannah V. Deane, Holly A. Concepcion, Holly DeCourcey, Ryan W. Logan, Joseph A. Seggio

**Affiliations:** ^1^Department of Biological Sciences, Bridgewater State University, Bridgewater, MA, United States; ^2^Department of Mathematics and Statistics, Boston University, Boston, MA, United States; ^3^Perelman School of Medicine, University of Pennsylvania, Philadelphia, PA, United States; ^4^Translational Neuroscience Program, Department of Psychiatry, University of Pittsburgh School of Medicine, Pittsburgh, PA, United States; ^5^Center for Systems Neurogenetics of Addiction, The Jackson Laboratory, Bar Harbor, ME, United States

**Keywords:** mouse model, circadian rhythm, strain difference, mice, constant light, running wheel

## Abstract

Previous studies have shown that exposure to circadian disruption produces negative effects on overall health and behavior. More recent studies illustrate that strain differences in the behavioral and physiological responses to circadian disruption exist, even if the strains have similar genetic backgrounds. As such, we investigated the effects of constant room-level light (LL) with running-wheel access on the behavior and physiology of male C57BL6/J from Jackson Laboratories and C57BL6/N from Charles River Laboratories mice. Mice were exposed to either a 12:12 light-dark (LD) cycle or LL and given either a standard home cage or a cage with a running-wheel. Following 6 weeks of LD or LL, their response to behavioral assays (open-field, light-dark box, novel object) and measures of metabolism were observed. Under standard LD, C57BL6/J mice exhibited increased locomotor activity and reduced exploratory behavior compared to C57BL6/N mice. In LL, C57BL6/J mice had greater period lengthening and increased anxiety, while C57BL6/N mice exhibited increased weight gain and no change in exploratory behavior. C57BL6/J mice also decreased exploration with running-wheel access while C57BL6/N mice did not. These results further demonstrate that C57BL/6 substrains exhibit different behavioral and physiological responses to circadian disruption and wheel-running access.

## Introduction

Numerous studies show that disrupted circadian rhythm can lead to abnormal behaviors. Individuals who are born with shifted circadian rhythms or genetic sleep disorders are commonly diagnosed with resulting depression and anxiety (McClung, 2007), suggesting common genetic substrates may contribute to both circadian rhythms and psychiatric disorders. Long-term circadian disruptions, such as constant light (LL) or models of ‘jet-lag’, can produce prolonged changes in behavior as animal models subjected to chronic circadian disruption can exhibit increased anxiety and depressive-like behaviors (Okuliarova et al., 2016). In addition to altering behavioral outcomes, circadian disruption can adversely affect metabolism and health. Shift-workers more commonly experience weight gain and obesity compared to regular work hour employees (Antunes et al., 2010). Subjecting animals to LL can significantly increase weight gain and insulin resistance and alter metabolic hormones (Coomans et al., 2013). Even dimmer light at night can produce negative health consequences (Fonken et al., 2010). The latest studies are also demonstrating links between psychiatric disorders and metabolic dysfunction, including increased risks for obesity and insulin resistance (Zuccoli et al., 2017; Penninx and Lange, 2018). A potential connection among circadian disruption, metabolic syndromes, and behavioral disorders is Brain-Derived Neurotropic Factor (BDNF). Altered or reduced BDNF is implicated in psychiatric conditions including increased anxiety (Colzato et al., 2011), poorer metabolic outcomes (Marosi and Mattson, 2014), and altered circadian function (Ikeno et al., 2016). Furthermore, TrkB (BDNF receptor) deficient mice exhibit altered circadian responses to light exposure (Allen et al., 2005).

One possible method of alleviating the negative physiological consequences of circadian disruption is through exercise. Recent studies in humans have shown that exercise can improve cardiovascular problems (Lim et al., 2015) and obesity (Kim et al., 2015) due to shift-work or jet-lag. These results may be due to how exercise hastens the resynchronization to new light-dark cycles (Eastman et al., 1995; Yamanaka et al., 2010). Running-wheel access also produces alterations to outcomes in commonly used assays that test explorative, anxiety-like, and learning behaviors in rodent studies. Wheel-running is a rewarding activity for rodents and usually produces anxiolytic effects (Greenwood et al., 2011; Roberts et al., 2012) and increases BDNF (Oliff et al., 1998), but the specific effects may be dependent upon the specific strain used, the environment, and/or experimental procedures used in the studies (Burghardt et al., 2004; Pietropaolo et al., 2006; Dubreucq et al., 2011). This study aims to uncover how the C57BL6/J (B6J) and C57BL6/N (B6N) mouse strains differ in their response to LL and wheel-running access. While genetically similar, the B6J and B6N substrains differ from each other behaviorally and physiologically (Banks et al., 2015; Sturm et al., 2015). Male B6J and B6N mice were exposed to constant indoor room-level lighting in either a standard cage or a cage with voluntary running-wheel access and assessed their metabolic and behavioral responses.

## Methods

### Animals and Circadian Rhythm Analysis

Thirty-eight male C57BL6/J (Jackson Laboratories, Bar Harbor, ME, USA) and C57BL6/N (Charles River Laboratories, Shrewsbury, MA, USA) mice were purchased at approximately 7 weeks of age. Mice were acclimated to a 12:12 h Light:Dark (LD) cycle for 1 week, consuming regular chow (LabDiet 5001, St. Louis, MO, USA) and water freely. The mice were individually housed in circadian rhythm monitoring cages, which either used continuous Infrared beam home-cage monitoring (IR), or with continuous access to running-wheels (RW, wheel diameter: 23 cm; StarrLife Sciences, Oakmont, PA, USA), as previously described (Nascimento et al., 2016). After acclimation, half of the mice in both genotypes and cage types were placed into constant room-level lighting (100 lux; LL), while the other half remained in 12:12 LD. Thus, there were eight total groups in a 2 × 2 × 2 setup: (1) B6J/IR/LD (*n* = 9); (2) B6J/IR/LL (*n* = 9); (3) B6J/RW/LD (*n* = 10); (4) B6J/RW/LL (*n* = 10); (5) B6N/IR/LD (*n* = 9); (6) B6N/IR/LL (*n* = 9); (7) B6N/RW/LD (*n* = 10); and (8) B6N/RW/LL (*n* = 10). Additionally, weekly measurements of body mass and food intake were recorded. All of the following assays and tissue collections listed below were conducted during the middle of each animal’s inactive time (approximately ZT or CT 6) and in the light, form the basis of comparison.

### Behavioral Assays

After 6 weeks of LL, explorative and learning and memory behaviors were assayed using the SmartCage™ software system, which uses automatic infra-red beam tracking of the locomotor activity of the animals (AfaSci Inc., Redwood City, CA, USA; Khroyan et al., 2012). The behavioral assays used in these sets of experiments were conducted using previously described methods including an open-field and light-dark box (L-D box) test (Hicks et al., 2016). A novel object recognition test was also conducted, using a 1-day protocol, modeled after Bevins and Besheer (2006); this assay is designed to test recognition memory. Initially, an individual mouse is placed into the open-field box with two of the same object (two rectangle Lego™ towers, same color, two blocks high, placed on opposite ends of the field, taped to the bottom of the box) and given 10 min to explore. The number of touches/sniffing of at least 1 s for both the left and right objects, as well as the amount of time spent on the right half of the box (regardless of interaction with the object), were manually recorded. After a 1-h delay where the animal was returned to their home cage, the animals were placed into the novel object arena again, except this time the right object was replaced with a new object (circular Lego™ tower of a different color, two blocks high, taped to the bottom) and given 3 min to explore. The number of touches/sniffing of at least once a second for both objects and the amount of time spent on the right side was recorded.

### BDNF Protein Levels

One week after the final behavioral assay (novel object), frontal lobe BDNF protein levels were assessed. After CO_2_ euthanasia, frontal lobe sections (approximating 1 mm^3^) were removed and immediately stored in −80°C. After storage, tissue homogenates were created in a cocktail containing Pierce IP Lysis buffer (Thermo Scientific, Rockford, IL, USA) and protease inhibitor (Halt Protease Inhibitor Single-Use Cocktail EDTA-Free 100×; Thermo Scientific) and 0.4 mL of protease/lysis cocktail was added for each sample. The samples were centrifuged at 4°C for 20 min at 2,000 *g*, and the supernatant tested in BDNF ELISA kits (Mouse BDNF PicoKine ELISA, Boster Biological Technology Co., Pleasanton, CA, USA), using a low target concentration (working dilution 1:2).

### Physiological Assays

Concurrent with brain section collection, whole blood was collected, allowed to clot, and then centrifuged at 4°C for 20 min at 2,000 *g*; the serum was used in free thyroxine ELISA Kits (MBS2508866, MyBioSource, San Diego, CA, USA). In addition, 50 mg liver samples were obtained and immediately stored in −80°C. After storage, the liver samples were homogenized in 300 μL of 5% Triton-X100 (Sigma-Aldrich Merck, St. Louis, MO, USA), centrifuged 4°C for 20 min at 2,000 *g*, and tested in EnzyChrom™ Triglyceride Assay Kits (Bioassay Systems, Hayward, CA, USA).

### Statistical Analyses

Circadian period (chi-square periodogram) and total daily locomotor activity were calculated using Clocklab (Actimetrics, Wilmette, IL, USA). Three-way ANOVAs with Tukey *Post hoc* pairwise comparisons for genotype, photoperiod, and home-cage type were used to uncover mean differences among all of the groups for the behavioral assays, physiological markers, and circadian locomotor activity.

## Results

### Circadian Locomotor Activity

Representative actograms are provided in Supplementary Figures S1, S2. All mice were able to entrain to the 12:12 LD cycle, and all mice placed into LL exhibited period lengthening. For overall activity, significant photoperiod (*F*_(1,59)_ = 8.65, *p* = 0.005, LD > LL), cage type (*F*_(1,59)_ = 19.30, *p* < 0.001, RW > IR), and strain (*F*_(1,59)_ = 4.39, *p* = 0.041, B6N < B6J) differences were observed, but there were no interactions (Supplementary Figure S3A). For circadian period, both strain/cycle (*F*_(1,59)_ = 9.80, *p* = 0.003) and cage/cycle interactions (*F*_(1,59)_ = 13.83, *p* = 0.001) were observed. In LL, B6Js had longer periods than B6Ns (*p* < 0.001). Additionally, while both IR and RW animals entrained successfully regardless of strain (*p* = 0.99), animals with running-wheels exhibited shorter circadian periods than animals without wheels in LL (*p* < 0.001; Supplementary Figure S3B).

### Open-Field

**Figure 1:**
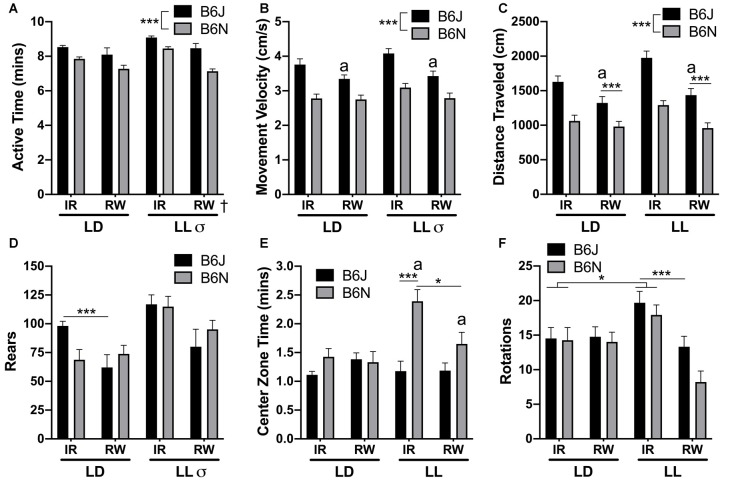
Open-field. **(A)** Photoperiod (LD < LL), cage (RW < IR), and strain (N < J) differences were observed for active time. **(B)** B6Js have greater velocity compared to B6Ns and reduce their velocity with RW compared to IR regardless of lighting condition [a = B6J/RW < B6J IR (in LD or LL)]. **(C)** B6J mice exhibited increased distance traveled compared to B6N, but RW reduces distance for B6Js only (although still significantly greater than B6N/RW groups), regardless of lighting condition [a = B6J/RW < B6J IR (in LD or LL)]. **(D)** LL increases rearing behavior in both strains but RW reduces it for B6Js only in LD. **(E)** B6N in LL exhibited the greatest center zone time amongst all other groups [a = B6N/LD < B6N/LD (in RW or IR)]. B6N mice with RW had lower center zone time than B6N in IR cages in LL. **(F)** In IR cages, LL increased total rotations compared to LD but RW reduced rearing due to LL, regardless of strain. ^†^Running-wheel difference, ^σ^LD vs. LL difference, *significantly different from each other at *p* < 0.05, ***p < 0.001. ^a^Denotes significant difference of the comparison previously described, at *p* < 0.05.

For Active Time, significant photoperiod (*F*_(1,68)_ = 4.87, *p* = 0.031, LD < LL), cage type (*F*_(1,68)_ = 22.36, *p* < 0.001, RW < IR), and strain (*F*_(1,68)_ = 30.96, *p* < 0.001, B6N < B6J) differences were observed (Figure 1A). LL exposure produced a difference in velocity regardless of cage type or strain (*F*_(1,68)_ = 4.08, *p* = 0.047, LD < LL). Strain differences (*F*_(1,68)_ = 74.05, *p* < 0.001, B6N < B6J) and a cage/strain interaction was uncovered for velocity (*F*_(1,68)_ = 3.96, *p* = 0.050). B6J mice with running-wheel access reduced their velocity through the open-field (*p* = 0.001); B6N had no such attenuation (*p* = 0.59; Figure 1B). Strain differences (*F*_(1,68)_ = 79.17, *p* < 0.001, B6N < B6J) and a cage/strain interaction was uncovered for Distance (*F*_(1,68)_ = 4.09, *p* = 0.047). B6J mice reduced their distance traveled when given access to a running wheel compared to their non-running counterparts (*p* < 0.001), but B6N mice exhibited a non-significant reduction with running wheel access (*p* < 0.067). Mice in the LD/IR groups had reduced distance compared to LL/IR animals (*p* = 0.008), but running-wheel access negated that difference (*p* = 0.94). This result is probably due to the fact that animals in LL/RW had attenuated distance traveled compared to LL/IR (*p* < 0.001; Figure 1C). Overall rearing was reduced in LD vs. LL (*F*_(1,68)_ = 14.69, *p* < 0.001). A cage/strain interaction was found (*F*_(1,68)_ = 4.59, *p* = 0.036), where the reduction of rearing in running-wheel cages was present for B6J (*p* < 0.001), but not B6N (*p* = 0.87; Figure 1D).

Two-way interactions for cycle/strain (*F*_(1,68)_ = 10.40, *p* = 0.002) and cage/strain (*F*_(1,68)_ = 6.48, *p* = 0.013) were observed for Center Zone time. In LD, no differences were found between B6N and B6J mice (*p* = 0.84), but under LL, B6N spent more time in the center than B6J (*p* < 0.001). While B6J mice spent equivalent times in the center of the open-field no matter the photoperiod (*p* = 0.97), B6N in LL spent more time in the center than B6N in LD (*p* = 0.001). Lastly, in IR B6J spend less time in the center zone than B6N (*p* < 0.001), but when given running-wheel access no differences were found (*p* = 0.50). The running-wheel led to a reduction in center zone time for B6N (*p* = 0.046), but not B6J (*p* = 0.80; Figure 1E). A cycle/cage interaction was observed for the total number of rotations (*F*_(1,68)_ = 14.46, *p* < 0.001); LL/RW had increased rotations compared to LD/RW (*p* < 0.001) and LD/IR had decreased rotations compared to LL/IR (*p* = 0.033; Figure 1F).

### Light-Dark Box

**Figure 2:**
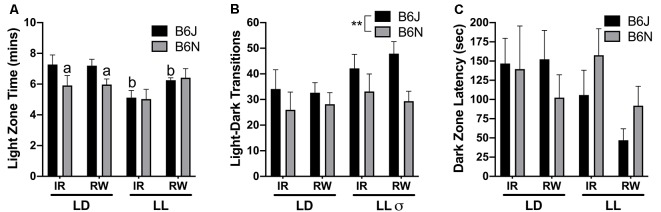
Light-dark box. **(A)** In LD but not LL, B6J mice exhibited increased light zone time compared to B6N, as indicated by the letter a. Light zone time is decreased in B6J/LL compared to B6J/LD, as indicated by the letter b. **(B)** Photoperiod (LD < LL) and strain (N < J) differences were observed for the number of transitions. **(C)** Dark Zone Latency is the time it takes for the mouse to make the first entry into the dark zone. No differences for dark zone latency were found among the groups. ^σ^LD vs. LL difference, **significantly different from each other at *p* < 0.01. a = B6N < B6J in LD, and b = B6J/LL < B6J/LD, at *p* < 0.05.

A cycle/strain interaction was uncovered for time spent in the Light Zone of the L-D box (*F*_(1,67)_ = 4.05, *p* = 0.049). In LD, B6N spent less time in the light zone than B6J (*p* = 0.038), but light zone time was not different between these strains in LL (*p* = 0.99). B6J mice decrease their light zone time under LL compared to LD controls (*p* = 0.004), but B6N have no change in light zone time no matter which photoperiod was given (*p* = 0.97; Figure 2A). Significant differences were present for the number of Transitions in the L-D box for photoperiod (*F*_(1,67)_ = 4.46, *p* = 0.040, LD < LL) and strain (*F*_(1,67)_ = 7.16, *p* = 0.010, B6N < B6J; Figure 2B). There were no significant differences amongst the groups for Latency until the first dark zone entry (all *p* > 0.08; Figure 2C).

### Novel Object

**Figure 3:**
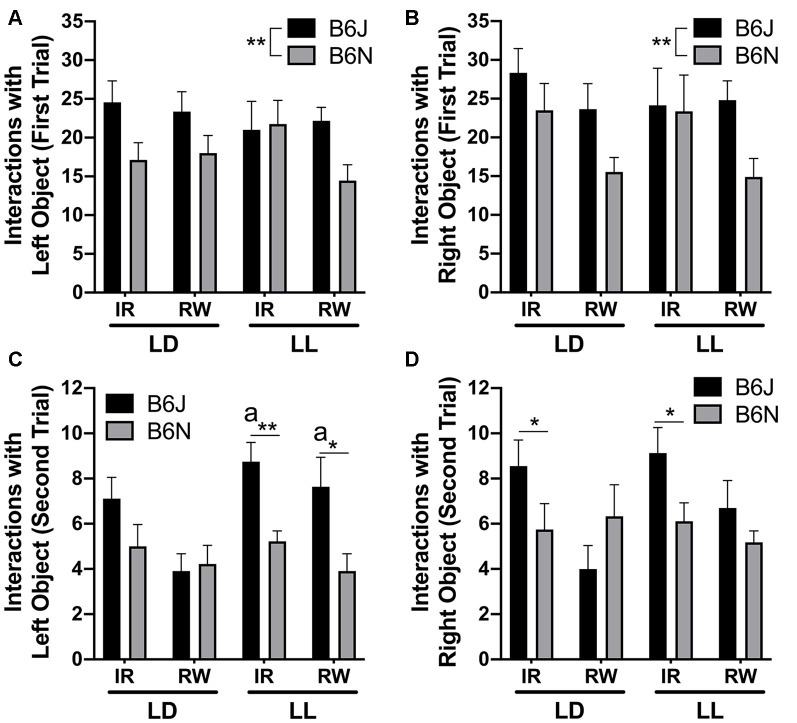
Novel object. **(A)** Initial phase interactions with left object (same) and **(B)** Initial phase interactions with right object (same) were increased in B6Js. **(C)** Second phase interactions with left object (old) were increased in B6J mice compared to B6N mice, regardless of cage type, as indicated by the letter “a.” **(D)** Second phase interactions with right object (new) were decreased in B6N mice compared to B6J mice, but only in standard caging (IR). *Significantly different from each other at *p* < 0.05, **p < 0.01. a = B6J/LL (IR or RW) > B6J/LD (IR or RW), at *p* < 0.05.

During the initial 10 min run, B6J mice exhibited increased novel object exploration for the left (*F*_(1,68)_ = 8.60, *p* = 0.005) and right objects (*F*_(1,68)_ = 7.38, *p* = 0.008; same type of object) compared to B6N (Figures 3A,B). The groups did not differ significantly regarding the amount of time spent in each zone (all *p* > 0.10). For the second 3 min phase (with the new object on the right side), there was a cycle/strain interaction for interactions on the left object (*F*_1, 68_ = 4.83, *p* = 0.031) and a cage/strain interaction for the right side (*F*_(1,68)_ = 5.28, *p* = 0.025). In LD, there were no differences between the strains (*p* = 0.74). Although no differences were found for B6J mice regarding the number of interactions between the old and new objects overall, in LL B6J had increased interactions with the old object than B6J in LD (*p* = 0.015) and B6N in LL (*p* < 0.001). For the new object, B6J exhibited increased interactions compared to B6N in IR (*p* = 0.040), but not so in RW (*p* = 0.098; Figures 3C,D). The groups did not differ significantly regarding the amount of time spent in each zone (all *p* > 0.10).

### BDNF

**Figure 4:**
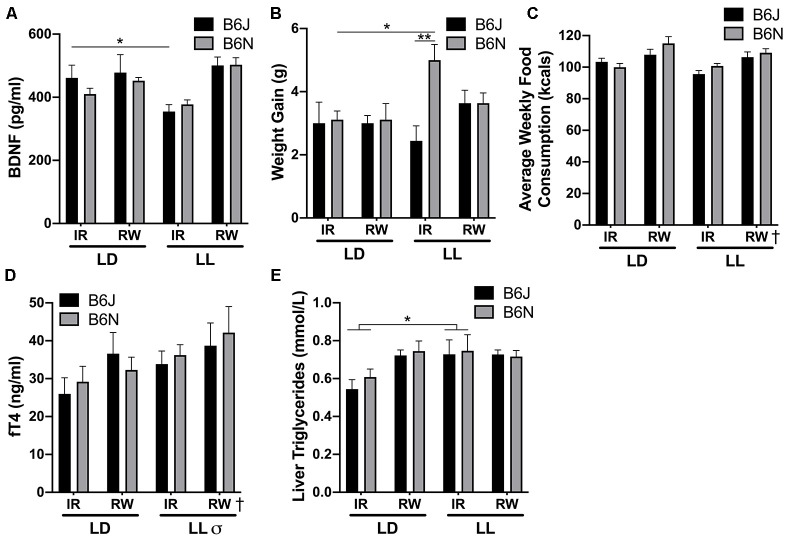
Physiological characteristics. **(A)** LL/IR for both strains reduced frontal lobe brain-derived neurotropic factor (BDNF) levels compared to other groups. **(B)** B6N/IR/LL gained the most weight compared to other groups. **(C)** RW increased food consumption regardless of strain or photoperiod. **(D)** LL and RW increased free Thyroxine in both strains. **(E)** Liver triglycerides were increased for both LL and RW mice of both strains. ^†^Running-wheel difference, ^σ^LD vs. LL difference, *significantly different from each other at *p* < 0.05, **p < 0.01.

No strain differences were found for frontal lobe BDNF levels (*F*_(1,56)_ = 0.43, *p* = 0.52). A cage/photoperiod interaction was uncovered (*F*_(1,56)_ = 7.39, *p* = 0.009). In IR, BDNF was significantly reduced in mice held in LL compared to LD (*p* = 0.050), but RW mice exhibited similar BDNF levels whether in LD or LL (*p* = 0.59; Figure 4A).

### Physiology

A significant three-way interaction (Genotype/Cage/ Photoperiod) was uncovered for weight gain (*F*_(1,68)_ = 5.03, *p* = 0.028). LL/B6N/IR mice experienced significant weight gain compared LD/B6N/IR (*p* = 0.046), but this weight gain was not observed for B6J mice (*p* = 0.98). LL/B6N/IR mice had increased weight gain compared to LL/B6J/IR (*p* = 0.002), but this was not found in LD (*p* = 0.99) or in LL/B6N/RW (*p* = 0.69; Figure 4B). Food consumption was significantly increased in animals with running-wheel access compared to IR animals regardless of genotype or photoperiod (*F*_(1,68)_ = 22.41, *p* < 0.001; Figure 4C). Both photoperiod (*F*_(1,61)_ = 5.62, *p* = 0.024, LD < LL) and running wheels (*F*_(1,61)_ = 4.67, *p* = 0.038, IR < RW) produced significant differences in fT4 serum levels, but there were no strain differences (*p* = 0.10; Figure 4D). A cage/photoperiod interaction was uncovered for liver triglycerides (*F*_(1,63)_ = 4.11, *p* = 0.049). IR/LD mice had significantly lower liver triglyceride levels compared to IR/LL (*p* = 0.011). Running-wheels had no effect on triglycerides in animals in LL (*p* = 0.68; Figure 4E).

## Discussion

We report behavioral and physiological differences in the response to LL and running-wheel access between male B6N and B6J mice, as well as several baseline differences. Overall, the B6J strain exhibited increased home-cage and running-wheel circadian locomotor activity compared to B6N mice. Additionally, B6Ns had shorter circadian periods in LL compared to B6J, which might be due to B6N mice having shorter endogenous free-running rhythms in constant darkness compared to B6J mice (Banks et al., 2015). A study which investigated the genomes of these two strains has identified several gene differences, some of which may regulate circadian clock function including *Adcy5* (influences locomotor activity levels), *Pmch* (mediates sleep and arousal), and *Crb1* (controls retina photoreceptor structure; Simon et al., 2013). In addition to the genetic differences between the two strains, the behavioral discrepancies seen in this study may also be due to the location of the breeding source and the providers of these mouse strains. A previous study using rats showed that Sprague-Dawley rats from three different vendors showed disparate levels of locomotor activity and different HPA axis and metabolic function (Pecoraro et al., 2006).

Male B6J mice have increased novelty-induced locomotor activity and exploration compared to B6N mice. B6N and B6J mice responded differently regarding their novelty-induced locomotor behavior in the open-field when given running-wheel access. B6J mice with running-wheels ran significantly more and when subsequently placed into an open-field, had significantly reduced locomotor activity compared to B6N mice in the same conditions. Other studies have also reported in B6J mice that running-wheel access (whether or not the wheel was locked prior to testing) reduces exploratory measures in the open-field (Duman et al., 2008; Salam et al., 2009; Fuss et al., 2010; Garrett et al., 2012; Morgan et al., 2018). Although B6N mice exhibited no such decrease in locomotor activity in the open-field with running-wheel access, these mice showed decreased exploratory behavior by reducing center zone time, as was seen in another study using B6N mice with access to running-wheels (Binder et al., 2004). The reduced locomotor activity, rearing, and center zone time in wheel-running rodents (as seen differences between the two strains in this study and in others previously cited) might lead to the conclusion that wheel-running increases anxiety-like behaviors compared to their sedentary counterparts. Interestingly, other studies show that elevated stress can produce similar reductions to exploration and increases to anxiety in the open field and light-dark box (Desbonnet et al., 2012; Monteiro et al., 2015), although it is worth noting that HPA axis function was not assessed in the current study. A recent study in which the two strains were given chronic corticosterone injections, reductions to their locomotor activity levels in the open-field occurred in both strains, but only the B6N mouse reduced their center zone time (Sturm et al., 2015). These results suggest that behavioral differences between these B6 substrains in response to access to running wheels may be partially due to baseline differences in anxiety, locomotor activity, and perhaps even, their behavioral stress response to novel environments.

We also uncovered the main strain difference in the anxiety-like behavioral responses to constant room-level light exposure in the L-D box. B6J mice might be more susceptible to the negative behavioral disruptions due to LL, as they decreased their time in the light zone when exposed to LL. Meanwhile, B6N did not decrease their light zone time and even spent more time in the center zone of the open-field when exposed to LL. Part of this difference may be due in part to B6N mice exhibiting a higher baseline anxiety-like behavior compared to B6J mice. Interestingly, previous studies using a variety of different inbred and outbred mouse strains have found that LL produces different effects on behavior depending upon the mouse strain or rat line. These studies studying explorative and anxiety-like behaviors under LL observe no differences in L-D box dark time, and/or elevated-(+) closed-arm time, which are the main measures of an anxiety-like state in these behavioral assays (Fonken et al., 2009; Roman and Karlsson, 2013; Tapia-Osorio et al., 2013). It is worth noting, however, that these aforementioned studies and others do report increased transitions and other measures of locomotor activity in both the L-D box and open-field when under LL (Marin et al., 2015). Nevertheless, other studies also report that non-B6J strains do not have the same alteration of novelty-induced locomotor activity when exposed to LL (Fujioka et al., 2011; Zhou et al., 2018) once again indicating a difference between the B6J strain and others. The difference baseline behavior between the two strains is also evident during the initial training during the novel object test where B6J mice exhibited increased novel object exploration during the initial training period than B6N mice. LL also affected object exploration differently between the two strains as B6J mice exhibited increased interactions with the old object during the second trial compared to controls, while no differences were found in B6N mice. On the other hand, it would seem that LL affects more depressive-like states behaviors regardless of the strain or species used (Fonken et al., 2009; Martynhak et al., 2011; Dimatelis et al., 2012; Roman and Karlsson, 2013; Tapia-Osorio et al., 2013; Marin et al., 2015; Zhou et al., 2018).

Interestingly, no baseline strain difference in BDNF was found and LL reduced BDNF levels in both substrains, a result which is similar to other studies which also illustrate the reduction in BDNF with circadian disruption (Fonken and Nelson, 2013; Ikeno et al., 2016). This result indicates that the substrain differences in behavioral responses under circadian disruptions found in this study may not be due to BDNF signaling despite its link to both modulating circadian function and other neurological behaviors. Wheel-running has been shown to produce increases to BDNF on its own particularly within the hippocampus in rodent models (Oliff et al., 1998; Stranahan et al., 2009). Although wheel-running on its own has been shown not to be sufficient in inducing increases to BDNF within the frontal lobe (Fuss et al., 2010), running-wheel access did improve the reduced BDNF in the frontal lobe for both strains when in LL, indicating that voluntary exercise may be useful in promoting neuronal health when exposed to circadian disruption regardless of strain or model.

B6N mice in LL without running-wheels experienced increased weight gain compared to their B6J counterparts in LL. Overall, B6N mice appear to be more sensitive to metabolic issues than B6J mice under certain conditions, including high-fat diet consumption (Podrini et al., 2013). Regardless of the previously listed strain differences found in their responses to continuous light exposure, this study provides additional evidence that LL can produce negative metabolic effects overall, including increased liver triglycerides and different fT4 levels, despite similar levels of food consumption. A recent study from our lab also reported altered fT4 and other hormone levels related to obesity and thyroid function in male CD-1 mice exposed to LL, even in the absence of weight gain or increased food consumption (Maroni et al., 2018). In summary, these results illustrate that circadian disruption can lead to significant changes in metabolic phenotypes even if a healthy diet is consumed. Meanwhile, access to a running-wheel improved some of the negative health issues caused by LL, by reducing the weight gain in B6N and improving BDNF levels, but did not reduce liver triglycerides. Running-wheel access, which can mimic voluntary exercise in rodent models, has been shown to relieve the negative health consequences due to circadian disruption in other studies (Fonken and Nelson, 2014; Nascimento et al., 2016). These results imply that exercise can counteract some, but not all, of the obesogenic and diabetic effects of circadian disruption and provide moderate improvement to overall health.

Still, it is worth noting that the mice in this study were individually housed rather than grouped housed and some studies have shown different behaviors in mice depending upon the housing (Bartolomucci et al., 2003; Febinger et al., 2014). Previous studies reported that male C57BL/6N mice singly or group-housed did not differ in their behavior or stress response (Arndt et al., 2009; Kamakura et al., 2016). Additionally, different male C57BL/6N mice within the grouped house may exhibit different behavior and hypothalamic *Crh* mRNA if they are subordinate within the group hierarchy compared to the others (Horii et al., 2017). Additionally, this study only investigated male mice, which were more widely used historically in studies assessing behavior and physiology. Female mice are known to exhibit different behavioral responses to a wide range of assays compared to males, including in the B6 substrains (Romeo et al., 2003; Gelineau et al., 2017).

In conclusion, numerous differences were found between the B6J and B6N strains of male mice for both behavior and physiology alone as well as in response to circadian disruption and running-wheel access. B6J mice exhibited increased novelty-induced and circadian locomotor activity compared to B6N mice. In LL, B6N mice exhibited increased weight gain but not increased anxiety, while B6J mice had no weight gain in LL but increased anxiety. B6J mice ran more on a running-wheel, exhibited a reduction in their novelty-induced activity, and had greater period lengthening in LL compared to B6N. Overall, B6N mice were more susceptible to the negative physiological effects of circadian dysregulation with higher baseline anxiety-like behaviors while B6Js were more susceptible to behavioral changes in response LL. As the C57BL/6 mouse is the most widely used mouse strain in the world used in a wide variety of research areas, these results imply that future studies will need to take into account the different behavioral and physiological responses to various stimuli between the different substrains of this mouse model.

## Data Availability Statement

The datasets generated for this study are available on request to the corresponding author.

## Ethics Statement

This study had the approval of Bridgewater State University’s Institutional Animal Care and Use Committee.

## Author Contributions

JS and RL designed the experiments and wrote the manuscript. KC, MM, HVD, HC, and HD performed all experiments and statistical analyses. All authors interpreted the data and approved the manuscript.

## Conflict of Interest

The authors declare that the research was conducted in the absence of any commercial or financial relationships that could be construed as a potential conflict of interest.

## Supplementary Material

FIGURE S1 Representative actograms for mice held in IR. **(A)** B6J/LD, **(B)** B6N/LD, **(C)** B6J/LL, **(D)** B6N/LL.

FIGURE S2 Representative actograms for mice held in RW. **(A)** B6J/LD, **(B)** B6N/LD, **(C)** B6J/LL, **(D)** B6N/LL.

FIGURE S3 Circadian home-cage locomotor activity. **(A)** Home-cage locomotor activity was higher in B6Js, LD, and RW animals, but no interactions were present. **(B)** LL produced period lengthening in both strains. RW reduced the period lengthening in LL. B6Js exhibited increased period lengthening compared to B6Ns in LL regardless of cage type. †: running-wheel difference, σ: LD vs. LL difference, *significantly different from each other at *p* < 0.05. a = B6J/LL > B6J/LD and b = B6N/LL > B6N/LD, at *p* < 0.05.
