# Supplementary figures and images for: Male C57BL6/N and C57BL6/J Mice Respond Differently to Constant Light and Running-Wheel Access
Source: Front Behav Neurosci. 2019 Dec 10;13:268. doi: 10.3389/fnbeh.2019.00268 (PMC6914853; doi:10.3389/fnbeh.2019.00268)

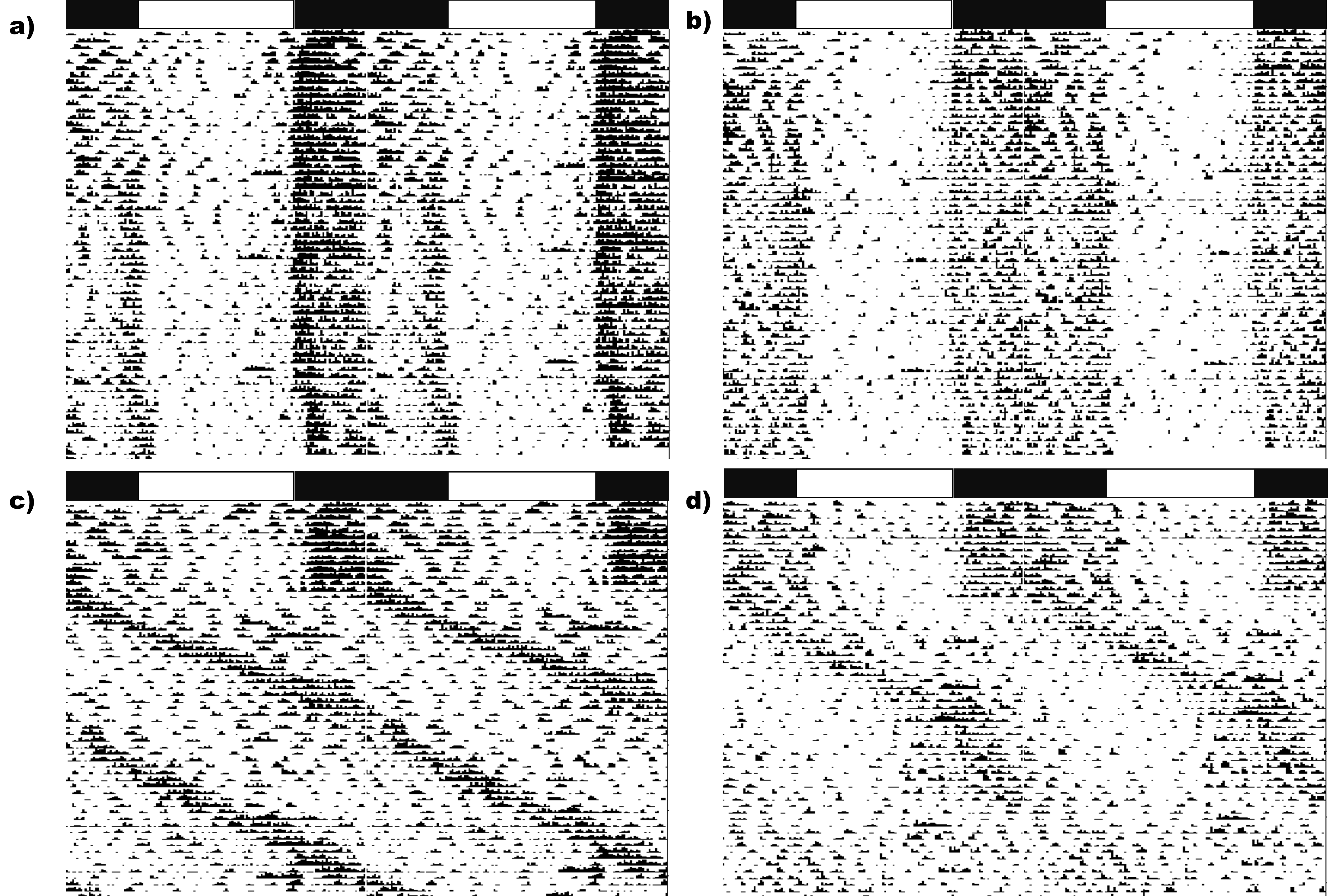

Supplement: FIGURE S1 — Representative actograms for mice held in IR. (A) B6J/LD, (B) B6N/LD, (C) B6J/LL, (D) B6N/LL. [file Image_1.TIF]

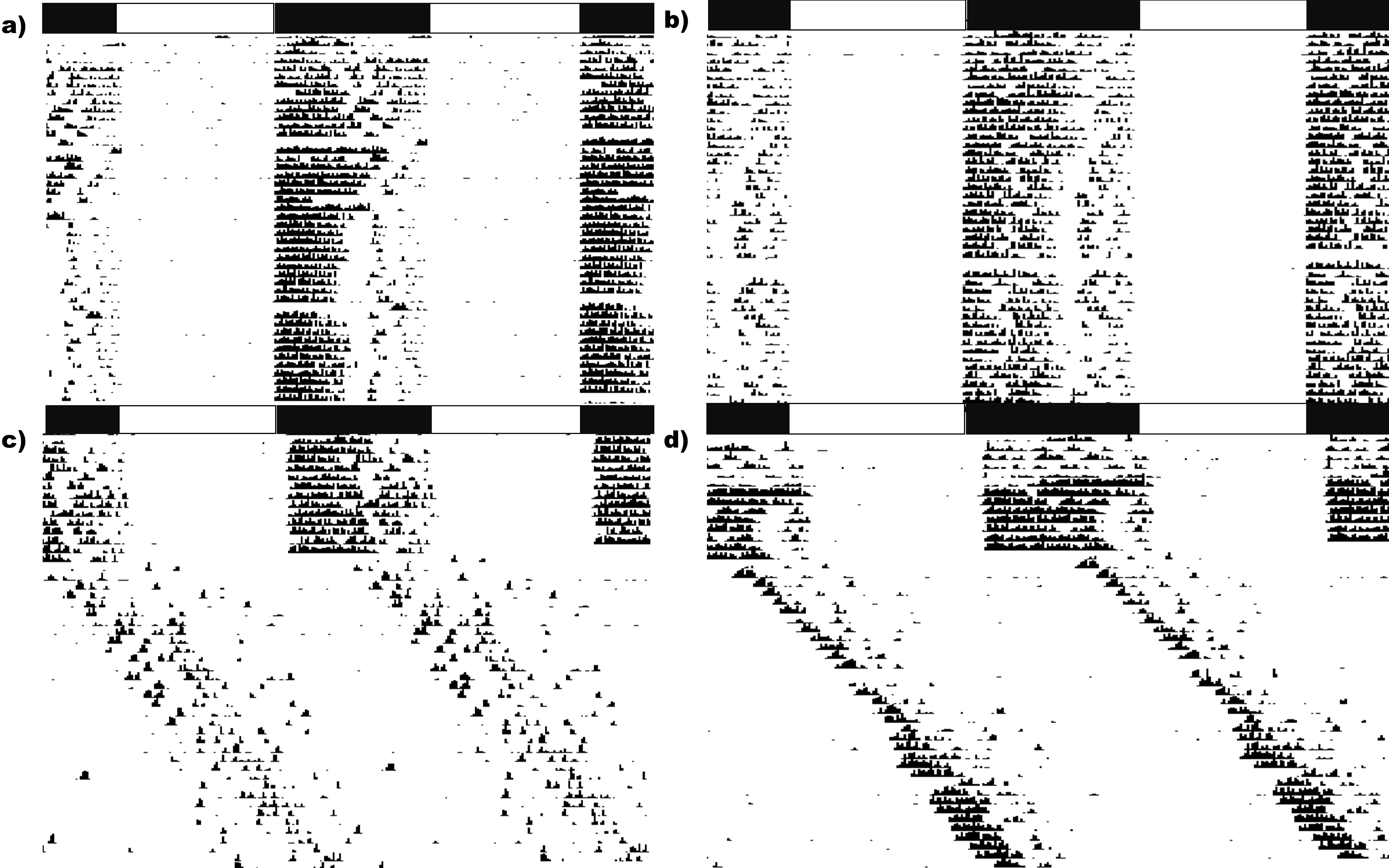

Supplement: FIGURE S2 — Representative actograms for mice held in RW. (A) B6J/LD, (B) B6N/LD, (C) B6J/LL, (D) B6N/LL. [file Image_2.TIF]

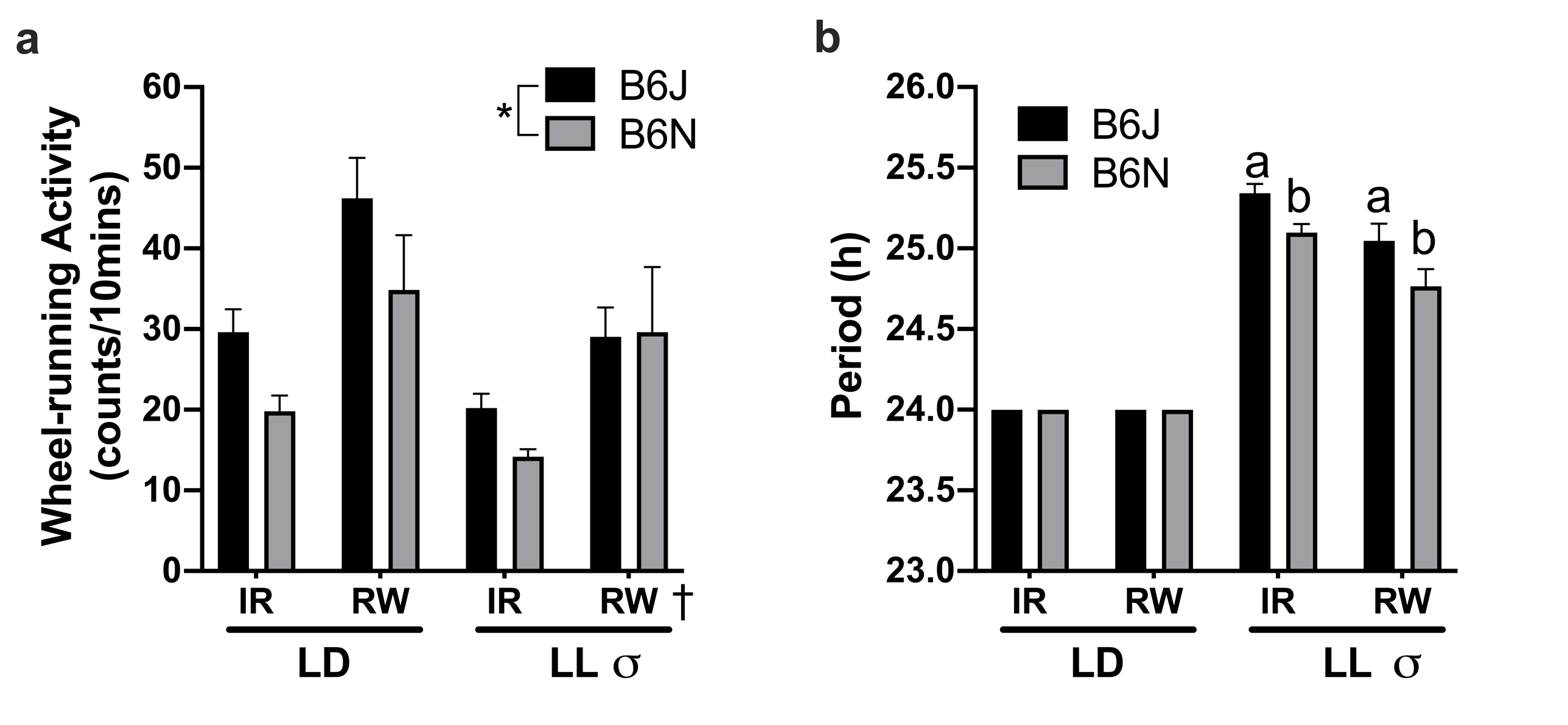

Supplement: FIGURE S3 — Circadian home-cage locomotor activity. (A) Home-cage locomotor activity was higher in B6Js, LD, and RW animals, but no interactions were present. (B) LL produced period lengthening in both strains. RW reduced the period lengthening in LL. B6Js exhibited increased period lengthening compared to B6Ns in LL regardless of cage type. †: running-wheel difference, σ: LD vs. LL difference, *significantly different from each other at p < 0.05. a = B6J/LL > B6J/LD and b = B6N/LL > B6N/LD, at p < 0.05. [file Image_3.tif]
